# Senescence and Stress Signaling Pathways in Corneal Cells After Nitrogen Mustard Injury

**DOI:** 10.3390/cells13232021

**Published:** 2024-12-06

**Authors:** Khandaker N. Anwar, Mohammad Soleimani, Mohammad Javad Ashraf, Amirhossein Moghtader, Raghuram Koganti, Seyyedehfatemeh Ghalibafan, Mahbod Baharnoori, Zohreh Arabpour, Kasra Cheraqpour, Aron M. Sebhat, Mansour Abtahi, Xincheng Yao, Mahmood Ghassemi, Ali R. Djalilian

**Affiliations:** 1 Department of Ophthalmology and Visual Sciences, University of Illinois Chicago, Chicago, IL 60612, USA; kanwar@uic.edu (K.N.A.); msolei2@uic.edu (M.S.); mjashraf1346@gmail.com (M.J.A.); moghtada@uic.edu (A.M.); rkogan3@uic.edu (R.K.); f.ghalibafanf@gmail.com (S.G.); sbahar2@uic.edu (M.B.); arabpourzohreh@gmail.com (Z.A.); cheraqpourk@gmail.com (K.C.); asebha2@uic.edu (A.M.S.); xcy@uic.edu (X.Y.); ghassemi@uic.edu (M.G.); 2 Department of Biomedical Engineering, University of Illinois Chicago, Chicago, IL 60607, USA; abtahi@uic.edu

**Keywords:** mustard, nitrogen mustard, mustard keratopathy, senescence, MAPK, MAPK inhibitor, BIRB796, ocular surface, cornea

## Abstract

Mustard gas keratopathy (MGK), a complication of exposure to sulfur mustard, is a blinding ocular surface disease involving key cellular pathways, including apoptosis, oxidative stress, and inflammation. Recent studies indicate that cellular senescence contributes to the pathophysiology of mustard gas toxicity. This study aimed to assess senescence and stress-related pathways—particularly mitogen-activated protein kinase (MAPK) signaling—in nitrogen mustard (NM)-induced corneal injury. In vitro, primary human corneal epithelial (P-HCECs), primary human corneal mesenchymal stromal cells (hcMSCs), and human corneal–limbal epithelial cell (HCLE) lines were exposed to varying concentrations of NM. The results demonstrated a dose-dependent increase in cellular senescence, characterized by reduced Ki67 expression, elevated p16, and p21 mRNA levels, as well as activation of the MAPK pathway activation. Treatment with a selective p38-MAPK inhibitor significantly reduced senescence markers and improved cell proliferation following exposure to NM. Overall, these studies indicate that NM exposure triggers cellular senescence and stress-related MAPK signaling, while p38-MAPK inhibition mitigates these effects, suggesting a potential therapeutic strategy.

## 1. Introduction

Mustard gas (MG) is a potent blistering and alkylating agent capable of causing severe chemical injuries. Initially developed in the 18th century, it gained notoriety during World War I as a low-cost chemical weapon with effects varying based on dosage and exposure time [1,2]. Despite being used as recently as 2017, no antidote or definitive treatment exists, and current management remains primarily supportive, aimed at mitigating exposure-related complications [3,4,5].

MG primarily targets the eyes, skin, and respiratory system, leading to a wide range of adverse effects. Early symptoms may include blistering of the skin, ocular irritation, and damage to the tracheobronchial mucosa [6,7]. Prolonged exposure can result in severe complications, such as mustard gas keratopathy (MGK), irreversible blindness, chronic laryngitis, pulmonary fibrosis, lung and skin cancers, respiratory failure, and even death [8,9].

Ocular toxicity affects up to 90% of individuals exposed to MG, with symptoms sometimes appearing months or even years post-exposure. These symptoms manifest in acute, chronic, or delayed forms, often persisting long after the initial exposure [1]. Acute symptoms—such as eye pain, burning, conjunctivitis, photophobia, and decreased vision—typically appear within 30 min and may resolve within 2 to 6 weeks [10]. Recovery outcomes vary, ranging from full resolution to the onset of chronic or latent MGK, where severe cases may lead to persistent epithelial defects, limbal stem cell deficiency, corneal neovascularization, and opacification [6,10,11,12]. Long-term complications such as keratitis and retinal dysfunction can appear 15 to 20 years post-exposure [8].

Although the exact pathophysiology of MG-induced damage remains unclear, multiple pathways are thought to contribute including oxidative stress, inflammation, cell death, and other signaling cascades [13,14,15]. Sulfur mustard (SM) specifically causes DNA cross-linking and single- and double-stranded breaks, leading to DNA damage and inhibition of cell division [16,17]. Depending on the affected cell type, SM exposure may induce apoptosis or necrosis, further exacerbated by reactive oxygen species (ROS) and reactive nitrogen species (RNS), as well as proteases released from damaged cells [17,18,19,20].

Cellular senescence has recently been recognized as a consequence of MG toxicity, as detected after sulfur analog exposure in dermal fibroblast and mesenchymal stem cell models [21,22,23]. Similarly, we were the first to report, using a murine model, that nitrogen mustard exposure induces dose-dependent senescence and fibrosis in the cornea, which can lead to late-onset ocular manifestation [24].

Senescent cells promote the release of pro-inflammatory cytokines into the extracellular space, a process known as the senescence-associated secretory phenotype (SASP) [25,26]. The chronic inflammation and accumulation of these senescent cells can impair wound healing, potentially contributing to the long-term or delayed effects of MG exposure [21,27].

The mitogen-activated protein kinase (MAPK) pathways play a crucial role in initiating cellular senescence [28]. Well-characterized MAPKs, such as ERK1/2 and p38 phosphorylate, regulate various proteins involved in cell survival, growth arrest, SASP secretion, apoptosis, and inflammation. These pathways serve as critical checkpoints within senescent cells, shaping their role in tissue damage and repair [28,29,30].

Building on insights from our recent murine model study [1], we have now conducted an in vitro investigation to further elucidate the molecular mechanisms underlying senescence signaling pathways in nitrogen mustard-induced corneal injury and to assess the impact of MAPK inhibition on mitigating the ocular effects resulting from NM.

## 2. Materials and Methods

### 2.1. Materials

Nitrogen mustard (NM), CelLytic, and all primers were purchased from MilliporeSigma (Rockville, MD, USA). All primary antibodies and Senescence-Associated β-galactosidase Staining Kit were purchased from Cell Signalling (Danvers, MA, USA), except for p16 (Abcam, Waltham, MA, USA), p21, and GAPDH (SCBT, Dallas, TX, USA). All secondary antibodies (HRP, FITC, and CY3) were purchased from Jackson ImmunoResearch Labs (West Grove, PA, USA). All Media (KSFM, Mem-alfa, and Tripley) and chemicals were purchased from Thermo Fisher Scientific (Carlsbad, CA, USA).

### 2.2. NM Preparation

Nitrogen mustard was tested at concentrations of 0.1 mM, 0.033 mM, and 0.01 mM. To prepare these concentrations, liquid NM (mechlorethamine hydrochloride), was aliquoted and stored at −80 °C. Before each experiment, an aliquot was thawed, diluted in PBS, and used immediately. Any remaining NM was discarded following the University of Illinois at Chicago (UIC) Environmental Health and Safety protocol.

### 2.3. Cells

Primary human corneal mesenchymal stromal/stem cells (hcMSCs), primary human corneal epithelial cells (P-HCECs), and human corneal–limbal epithelial (HCLE) cell lines were used following our previously published protocols [31,32]. The hcMSCs and P-HCECs were derived from healthy human cadaver corneas, with donors having a history of cancer and diabetes excluded. IRB approval was not required per UIC guidelines for the Protection of Research Subjects, as the corneas were obtained from deceased donors and no identifiable information was provided to us [33].

After retrieval from storage media, the cadaver corneas were rinsed three times with sterile PBS containing 1% antibiotic–antimycotic and 1% gentamicin. The central corneas were excised using an 8 mm trephine. The remaining corneoscleral rim was divided into six pieces for further processing to isolate corneal MSCs or epithelial cells. The hcMSCs were cultured using a modified protocol based on validated methodologies [31,32]. The donor corneas were between one and two weeks post-mortem, with donor ages ranging from 9 to 32 years.

Corneal tissue pieces were cultured in 6-well plates pre-coated with 1% collagen type I with the epithelial surface placed face-down. The culture medium consisted of MEM-α supplemented with 10% fetal bovine serum, 1% antibiotic–antimycotic, L-glutamine, and non-essential amino acids (NEAA) (all from Thermo Fisher Scientific). Cultures were incubated at 37 °C in a humidified atmosphere containing 5% CO_2_.

To prevent explant detachment, 200 μL of media was added on top of each piece during the initial days. Once the explants adhered, the culture media was gently replaced every alternate day. After 7 days, the explants were removed with fine-tip iris forceps, and the remaining cells (a mixture of MSCs and epithelial cells, Passage 0) were detached using TrypLE.

The isolated cells were expanded in culture with media changes every 2 to 3 days. By Passage 3, only the MSCs continued proliferating. For this study, hcMSCs from Passages 3 to 8 were utilized.

Epithelial cell lines were derived by culturing limbal fragments under conditions similar to hcMSCs but in Epix media (KSFM supplemented with EGF, BPE, 1% antibiotic -antimycotic, Y-27632 (5 μM), A8302 (3 μM), and isoprenaline (6 μM) [34].

The HCLE cell line, generously provided by Ilene Gipson and Pablo Argueso (Schepens Eye Research Institute, Massachusetts Eye & Ear, and Harvard Medical School, Boston, MA, USA), was cultured in KSFM supplemented with EGF, BPE, and 1% antibiotic–antimycotic [35].

### 2.4. NM Exposure

To establish an in vitro model, cells were exposed to non-toxic concentrations of NM, ensuring partial survival. This approach simulated the in vivo environment, where most cells recover from acute injury but subsequently progress toward subtle, long-lasting pathology, ultimately leading to tissue impairment.

Nitrogen mustard was applied at concentrations of 0.1 mM, 0.05, 0.033 mM, and 0.01 mM to corneal MSCs and epithelial cells for two hours. Lower concentrations of 0.015 mM, 0.004 mM, 0.002 mM, and 0.001 mM were used for HCLE cells. Cell viability was measured using live/dead and MTT assays.

Fresh media were added following the washing procedure, and cultures were monitored for two to three weeks, with media replenished every 48 to 72 h. Morphological changes were tracked using brightfield microscopy, and senescent cells were identified by SA-β-gal staining.

### 2.5. Western Blot

Protein expression was determined using Western blots, as previously described [36]. Briefly, cells were lysed with CelLytic, and after measuring protein concentration using Bicinchoninic acid (BCA) assay, equal amounts of each sample were mixed with 4X NuPAGE LDS sample buffer (Thermo Fisher Scientific), denatured by heating at 75 °C for 10 min, and subjected to electrophoresis on NuPAGE 4–12% Bis–Tris gel (Thermo Fisher Scientific). After gel electrophoresis, the proteins were transferred to polyvinylidene fluoride membranes using the iBlot gel transfer (Thermo Fisher Scientific). The membranes were then incubated in 5% bovine serum albumin in tris-buffered saline with 0.01% Tween 20 (TBST) for 1 h, followed by incubation with primary antibody while shaking at 4 °C overnight. After washing with TBST and incubation with the respective horseradish peroxidase-conjugated secondary antibodies for 1 h at room temperature, protein bands were visualized using the SuperSignal West Femto Maximum Sensitivity Substrate (Thermo Fisher Scientific) with an ImageQuant LAS 4000 Biomolecular Imager (GE Healthcare Life Sciences, Marlborough, MA, USA). The results were quantified using Image J software, version 1.X.

### 2.6. Real-Time PCR

RNA expression was determined using polymerase chain reaction (PCR) as previously described [37]. Briefly, total RNA was extracted from treated cells using the GeneJET RNA kit (Thermo Fisher Scientific) according to the manufacturer’s protocol. After spectrophotometric assessment for quality and concentration (Nanodrop ND-1000; Thermo Fisher Scientific), complementary DNAs (cDNAs) were generated using the High Capacity cDNA Reverse Transcription Kit (Applied Biosystems, Carlsbad, CA, USA) according to the manufacturer’s protocol. For each reaction, 500 ng of total RNA was used. The quantitative PCR reactions were carried out in triplicate with Fast SYBER Green Master Mix (Applied Biosystems, Carlsbad, CA, USA) in a total volume of 10 μL, using universal thermal cycling conditions of 10 min at 90 °C and 10 s at 95 °C, followed by 40 cycles of 95 °C for 15 s and 60 °C for 1 min. Glyceraldehyde 3-phosphate dehydrogenase (GAPDH) and cytochrome C oxidase (COX1) were used as housekeeping genes, and geomean values were taken as references. The PCR data were analyzed using the comparative CT method. Briefly, the ΔΔCT is calculated by ΔΔCT = ΔCTq − ΔCTcb. ΔCTq is the difference in the CT of a gene of interest and a reference gene, and ΔCTcb is the difference in the CT of a gene of interest and a reference gene. The relative mRNA expression was determined using the 2^−ΔΔCT^ method and reported as a relative fold expression over the corresponding control values. The qPCR was performed in triplicate for each sample, and the average of at least three independent experiments was used [11].

### 2.7. Immunofluorescence Study

The cells were grown on collagen-coated chamber slides (Thermo Fisher Scientific) according to Abcam immunostaining protocol with modification [38]. Briefly, the cells were fixed with 4% paraformaldehyde at room temperature for 15 min. After washing with PBS, they were incubated with PBS containing 0.3% Triton X-100 (Sigma-Aldrich, Burlington, MA, USA) for 1 h at room temperature, followed by blocking with PBS containing 1% BSA and 0.3% Triton X-100 for 1 h at room temperature. The samples were incubated overnight at 4 °C with the primary antibody. After washing three times with TBST, the secondary antibody was applied for 1 h at room temperature, followed by counterstaining with 4′,6-diamidino-2-phenylindole (DAPI).

Imaging was performed with the same light intensity and exposure for all samples, using a confocal microscope (LSM 710, Carl Zeiss, Cambridge, UK). The results were analyzed with ImageJ (NIH) and were standardized based on the untreated intensity. The average of six random sections was taken for analysis.

### 2.8. Proliferative Capacity and Senescence Regulation

The proliferative capacity of hcMSC was evaluated using Ki-67 staining, following the immunofluorescence assay protocol (Abcam). Similarly, the effect of NM on cell cycle arrest, a hallmark of senescence, was assessed by quantifying the mRNA expression of p16 and p21. Immunofluorescent microscopy, utilizing antibodies against p16 and p21, was performed to further investigate these markers. Control cells, matched for passage number and media but unexposed to NM, were included for accurate comparisons.

### 2.9. MAPK Signaling Evaluation

The levels of p38, phosphorylated p38, p44/42 (ERK1/2), phosphorylated p44/42, SAPK/JNK, and phosphorylated SAPK/JNK—key indicators of the MAPK pathway—were compared between senescent and unexposed control groups via Western blotting.

To evaluate the efficacy of a MAPK inhibitor in counteracting or mitigating NM-induced cellular senescence, BIRB 796, a selective inhibitor of p38 mitogen-activated protein kinase (p38-MAPK), was chosen due to its established therapeutic effects in treating inflammatory and autoimmune conditions [39]. Cells were treated with BIRB 796 (0.01 μM and 0.1 μM) two hours after exposure to NM (0.1 mM).

Control groups comprised unexposed cells and NM-treated cells without the MAPK inhibitor. Cell counts were conducted across six high-power fields (HPF), and the average number of cells per section was computed for analysis.

### 2.10. β-Gal Staining Assessment

Senescent cells commonly express SA-β-Gal, reflecting increased lysosomal biogenesis and autophagy [40,41]. To identify senescent cells in our study, we employed a computer-assisted color segmentation method to quantify the β-gal staining ratio, utilizing the OpenCV library.

The analysis commenced with image preprocessing to enhance quality and reduce noise, followed by conversion to the hue saturation value (HSV) color space to improve color representation. Subsequently, thresholding algorithms were applied to differentiate β-gal-stained regions from adjacent cellular areas.

The total area of β-gal staining was quantified by summing the pixels within the segmented regions, and this value was divided by the total cellular staining area to determine the β-gal staining ratio.

### 2.11. Statistical Analysis

Statistical analyses were performed using SPSS (version 29.0.2.0, IBM Corp., Armonk, NY, USA) and GraphPad Prism (version 10.3.1, Dotmatics, Boston, MA, USA), with results presented as mean (M) ± standard deviation (SD). An independent samples *t*-test was utilized to determine significance, with *p* < 0.05 set as the threshold. For comparisons involving more than two groups, one-way ANOVA followed by Tukey’s post hoc correction was utilized to determine significance, with *p* < 0.05 set as the threshold.

## 3. Results

### 3.1. Morphological Changes in Human Corneal Cells

**Figure 1:**
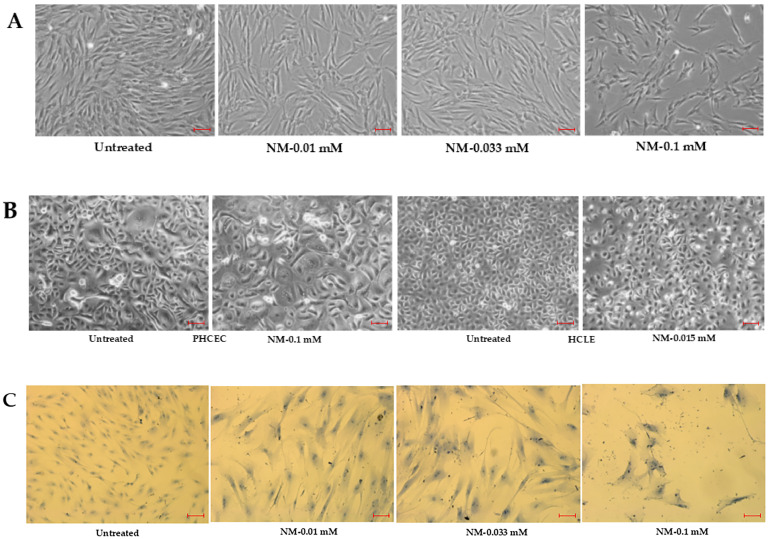
Phase-contrast microscopy images showing significant morphological changes in human corneal mesenchymal stromal cells (hcMSCs) treated with nitrogen mustard at concentrations of 0.1, 0.03, and 0.01 mM after three days (**A**). Notable morphological alterations in primary human corneal epithelial cells (HCECs) treated with nitrogen mustard at 0.1 and 0.015 mM are observed after two days (**B**). Giemsa staining further illustrates significant morphological changes in hcMSCs treated with nitrogen mustard at 0.1, 0.03, and 0.01 mM after one week (**C**) (scale bar (red): 100 μm).

Exposure to NM at concentrations of 0.1, 0.03, and 0.01 mM induced significant elongation and flattening with increased cell sizes in both hcMSCs and P-HCECs compared to controls. Additionally, a dose-dependent reduction in cell number was observed. These morphological changes were evident across various time points, including 24, 48, and 72 h, as well as one-week post-exposure, as captured by phase-contrast microscopy (Figure 1A,B) and Giemsa staining (Figure 1C). Phase-contrast images (Figure 1A,B) demonstrate progressive changes in cell morphology and density. Giemsa staining highlights structural alterations, including disrupted cell arrangements, loss of bipolar spindle-shaped morphology and enlargement, particularly in hcMSCs treated with 0.1 mM NM after one week. These findings indicate that NM exposure adversely affects cell morphology, viability, and potentially vascular integrity in vitro, as inferred from the altered cellular organization and density.

### 3.2. Proliferation-Related Gene Expression

Exposure to NM resulted in a significant reduction in the expression of proliferation-related genes in human corneal mesenchymal stem cells. Ki-67 mRNA and protein levels, key markers of cellular proliferation, were significantly reduced in a dose-dependent manner by 0.1 mM and 0.033 mM NM, leading to up to a fivefold decrease compared to controls.

**Figure 2:**
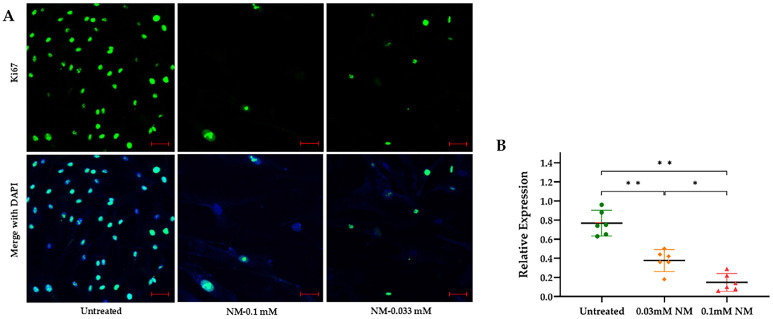
Immunofluorescence staining demonstrates a dose-dependent reduction in Ki-67 expression in human corneal mesenchymal stem cell (hcMSC) cultures, observed 2.5 weeks post-exposure to nitrogen mustard (NM) (scale bar (red): 50 μm) (**A**). Quantitative analysis reveals a significant decline in Ki-67levels in NM-treated cells at varying concentrations compared to untreated controls, with 95% confidence intervals (* *p* < 0.01; ** *p* < 0.001) (**B**). Ki-67 (green), DAPI (blue).

A one-way ANOVA revealed a significant effect of NM treatment on the relative expression of Ki67 within the cells, *p* < 0.001. Tukey’s post hoc tests indicated that the control untreated group exhibited a significantly higher relative expression compared to both the 0.03 mM NM treatment group (*p* < 0.001) and the 0.1 mM NM treatment group (*p* < 0.001). Furthermore, the 0.03 mM NM treatment group demonstrated a significantly higher relative expression than the 0.1 mM NM treatment group (*p* < 0.01). Notably, this suppression persisted for up to 2.5 weeks in human corneal mesenchymal stem cell (hcMSC) cultures (Figure 2).

### 3.3. Activation of Senescence Markers

**Figure 3:**
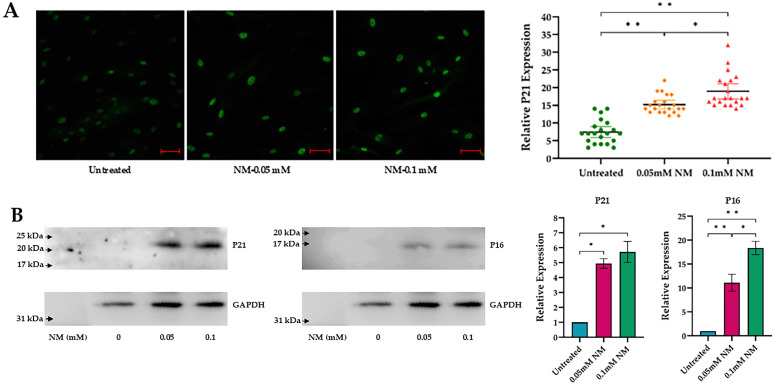
Expression of senescence markers in human corneal mesenchymal stem cells (hcMSCs) and primary human corneal epithelial cells (P-HCECs) following exposure to 0.05 and 0.1 mM nitrogen mustard (NM). Fluorescence staining illustrates the significant increase in p21-positive cells in hcMSCs two and a half weeks post-exposure to varying concentrations of NM, accompanied by 95% confidence intervals (scale bar: 50 μm) (* *p* < 0.01; ** *p* < 0.001) (**A**). Western blot analysis shows the detection of p16 and p21 levels in P-HCECs after one week of NM treatment, with glyceraldehyde 3-phosphate dehydrogenase (GAPDH) used as a housekeeping gene for normalizing protein expression levels, with standard deviation (P21 (green): * *p* < 0.001; P16: * *p* < 0.01, ** *p* < 0.001) (**B**).

To evaluate the expression of senescence markers at the protein level, hcMSCs and P-HCECs were treated with NM at concentrations of 0.05 and 0.1 mM. Confocal microscopy revealed a significant increase in p21-positive cells in hcMSCs after two and a half weeks. A one-way ANOVA indicated a substantial effect of NM treatment on the relative expression of p21 across both cell types, *p* < 0.001. Tukey’s post hoc test showed that the untreated control group exhibited lower relative expression compared to both the 0.05 mM NM treatment group (*p* < 0.001) and the 0.1 mM NM treatment group (*p* < 0.001). Additionally, the 0.05 mM NM treatment group also had significantly lower relative expression compared to the 0.1 mM NM treatment group (*p* < 0.01) (Figure 3A).

Western blot analyses demonstrated a significant, dose-dependent increase in the senescence markers p16 and p21 in P-HCECs after one week of NM treatment. A one-way ANOVA revealed a significant effect of NM treatment on the relative expression of p21, *p* < 0.001, confirming the findings observed in hcMSCs. Tukey’s post hoc test indicated that the untreated control group had lower relative expression than the 0.05 mM NM treatment group (*p* < 0.001) and the 0.1 mM NM treatment group (*p* < 0.001). The 0.05 mM NM treatment group also exhibited lower relative expression compared to the 0.1 mM NM treatment group (*p* < 0.01) (Figure 3B).

**Figure 4:**
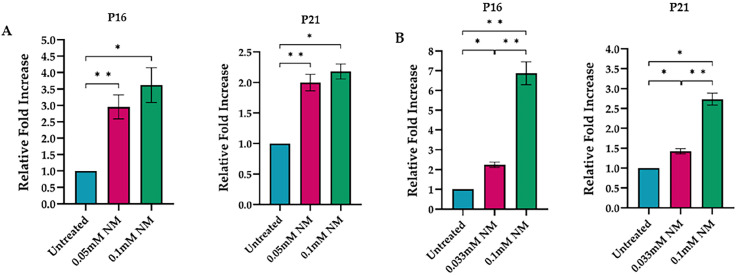
mRNA expression of p16 and p21 in human corneal mesenchymal stem cells (hcMSCs) and primary human corneal epithelial cells (P-HCECs) after exposure to nitrogen mustard (NM). The relative fold increase was assessed in hcMSCs three days after NM exposure, with standard deviation (P16: * *p* < 0.05, ** *p* < 0.001; P21: * *p* < 0.01, ** *p* < 0.001) (**A**). The relative fold increase was also assessed in P-HCECs five days after NM exposure, with standard deviation (* *p* < 0.01; ** *p* < 0.001) (**B**).

Further examination revealed that NM exposure (0.1 and 0.01 mM) resulted in a twofold increase in p16 mRNA and a threefold rise in p21 mRNA in both hcMSCs and P-HCECs after three days. Protein levels of p16 and p21 were elevated five days post-exposure, indicating robust activation of senescence pathways. An independent samples *t*-test confirmed that the untreated control group had significantly lower relative fold increases in p16 expression compared to the 0.05 mM NM-treated group, *p* < 0.001. Similarly, the control group exhibited a significantly reduced fold increase in p21 expression relative to the 0.1 mM NM-treated group, *p* < 0.01 (Figure 4A).

In P-HCECs, senescence markers p16 and p21 were significantly increased compared to untreated cells. An independent samples *t*-test indicated that the control group had a significantly lower relative fold increase in p16 expression compared to the 0.033 mM NM-treated group, *p* < 0.01. The control group also exhibited a significantly lower fold increase in p21 expression compared to the 0.1 mM NM-treated group, *p* < 0.001. Additionally, the 0.033 mM NM-treated group demonstrated a significantly lower relative fold increase than the 0.1 mM NM-treated group, *p* < 0.001 (Figure 4B).

**Figure 5:**
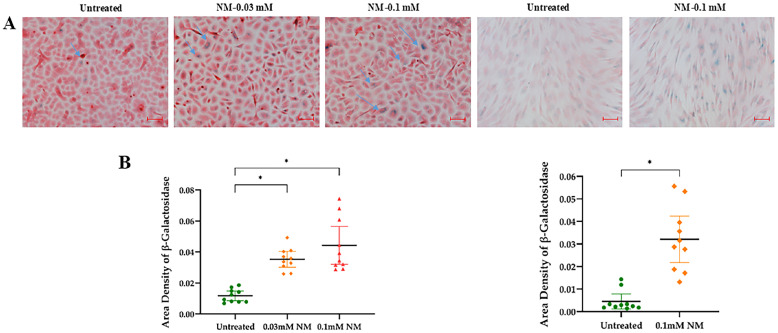
Representative images of SA-β-gal staining showing the effects of nitrogen mustard (NM) in different groups, including unexposed cells and 0.03 mM and 0.1 mM NM-exposed cells after 48 h of treatment in primary human corneal epithelial cells (P-HCECs), and 0.1 mM NM-exposed in human corneal mesenchymal stromal cells (hcMSCs) Blue arrows indicate epithelial cells stained with SA-β-gal (scale bar: 50 μm) (**A**). Quantitative analysis of SA-β-gal-positive cells revealed that the untreated control group had a significantly lower area density of β-galactosidase compared to both the 0.1 mM NM treatment group and the 0.03 mM NM treatment group in P-HCECs and to the 0.1 mM NM treatment group in hcMSCs, with 95% confidence intervals shown (* *p* < 0.001) (**B**).

The senescence effect of NM on hcMSCs was also assessed using senescence-associated β-galactosidase (SA-β-gal) staining. A higher number of blue-stained cells were observed in NM-treated groups compared to vehicle-treated controls after 48 h. The untreated control group had a significantly lower β-galactosidase area density compared to the 0.1 mM NM-treated group, *p* < 0.001. Similarly, the untreated group exhibited lower β-galactosidase area density than the 0.03 mM NM group, *p* < 0.001. The 0.25 mM NM group was excluded from analysis due to high toxicity, as no cells remained after 48 h (Figure 5).

### 3.4. Activation of MAPK Pathways

**Figure 6:**
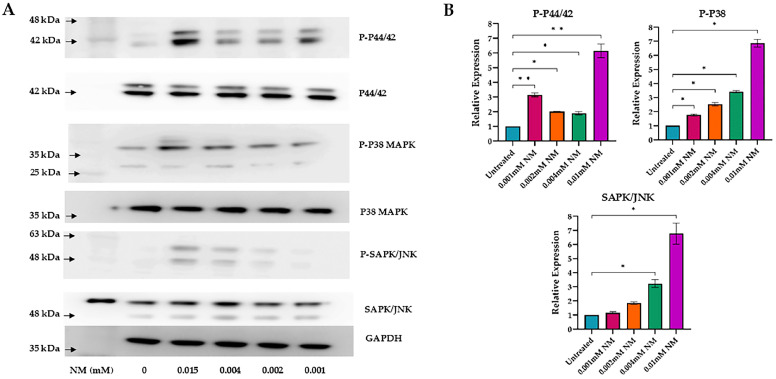
Western blot analysis of MAPK protein expression in human corneal mesenchymal stem cells (hcMSCs) and human corneal–limbal epithelial (HCLE) cells following exposure to varying concentrations of nitrogen mustard (NM). The results demonstrate a significant dose-dependent increase in the phosphorylation levels of MAPK proteins, including p-p44/42, p-p38, and p-SAPK/JNK (**A**). Quantitative analysis of the protein expression was conducted using one-way ANOVA and Tukey’s post hoc test, with standard deviation shown (P-P44/42: * *p* < 0.01, ** *p* < 0.001; P-P38 and SAPK/JNK: * *p* < 0.001) (**B**). Arrow (blue) showing senescent cells (green), scale bar (red).

To explore the relationship between NM and MAPK activation, we exposed HCLE cells with NM at increasing concentrations and measured the levels P-P38,P38,P-P44/42,P44/42, P-SAPK/JNK and SAPK/JNK via western blotting. We observed a dose-dependent increase in the phosphorylation of MAPK pro-inflammatory mediators (Figure 6).

### 3.5. MAPK Inhibition

The inhibition of p38 MAPK by BIRB796 significantly reduced NM-induced phosphorylation in HCLE cells over 20 h. This reduction was dose-dependent, with higher concentrations of BIRB796 resulting in a progressively greater decline in p38 phosphorylation.

**Figure 7:**
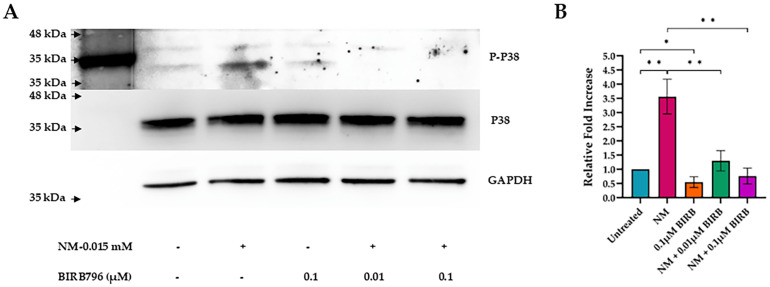
BIRB796, a specific inhibitor of p38 mitogen-activated protein kinase (MAPK), significantly reduced nitrogen mustard (NM)-induced phosphorylation of p38 in a dose-dependent manner in human corneal limbal epithelial (HCLE) cells after 20 h. Phosphorylated p38 (P-p38) protein expression was detected via Western blotting (**A**) and followed by a quantitative analysis of the protein expression with standard deviation shown (* *p* < 0.01; ** *p* < 0.001) (**B**). Arrow (black) showing Markers band.

A one-way ANOVA revealed a significant effect of NM and BIRB796 treatment on relative fold increase, *p* < 0.001. Tukey’s post hoc test indicated that the control untreated group had a lower relative fold increase than the 15 µM NM treatment group, *p* < 0.001. Additionally, the 15 µM NM treatment group exhibited a higher relative fold increase compared to the 15 µM NM with 0.01 µM BIRB treatment group, *p* < 0.001, and the 15 µM NM with 0.1 µM BIRB796 treatment group, *p* < 0.001. An independent samples *t*-test demonstrated that the control untreated group had a higher relative fold increase than the 0.1 µM BIRB796 treatment group, *p* < 0.01 (Figure 7).

**Figure 8:**
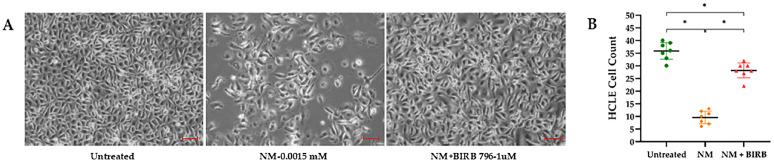
Phase-contrast microscopy images of human corneal limbal epithelial (HCLE) cells captured 2 days post-exposure to nitrogen mustard (NM), either alone or in combination with the MAPK inhibitor BIRB796. The images illustrate the characteristic features of cellular senescence. NM-treated cells exhibited flattened morphology and increased cell size compared to untreated and BIRB796-treated cells, which maintained a morphology comparable to that of the untreated cells (scale bar: 100 μm) (**A**). Cell counts of HCLE cells following various treatment exposures are presented as the mean number of cells per high-power field (HPF) across seven sections, with 95% confidence intervals (* *p* < 0.001) (**B**).

In our study, treatment with the MAPK inhibitor BIRB796 (1 µM) significantly enhanced cell proliferation. After two days of NM exposure (0.01 mM), HCLE cells exhibited noticeable morphological changes, including a substantial decrease in cell numbers compared to controls. The addition of BIRB796 to NM-treated cells effectively restored proliferation to levels similar to those of unexposed samples (Figure 8).

**Figure 9:**
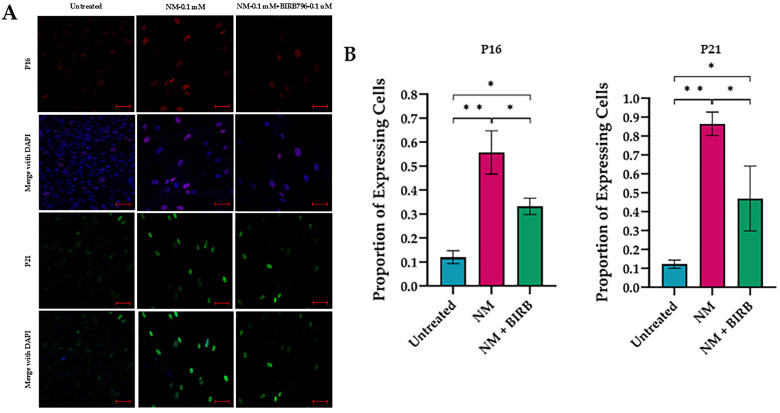
Immunofluorescence detection of p16 and p21 in human corneal mesenchymal stem cells (hcMSCs) 5 days after exposure to 0.1 mM nitrogen mustard (NM) and a combination of NM and BIRB796 (0.1 µM) (scale bar: 50 μm) P16 (Red), DAPI (Blue). P21 (Green) (**A**). Corresponding quantitative analysis, with standard deviation (* *p* < 0.01; ** *p* < 0.001) (**B**).

To investigate the potential of p38 MAPK inhibition in hcMSCs for reducing cellular senescence, we found that treatment with BIRB796 (0.1 µM) significantly reduced the number of senescent cells expressing p16 and p21. This reduction was confirmed by immunofluorescence assays after five days of exposure to NM (0.1 mM) and the combination of NM with BIRB796 (0.1 µM) (Figure 9).

**Figure 10:**
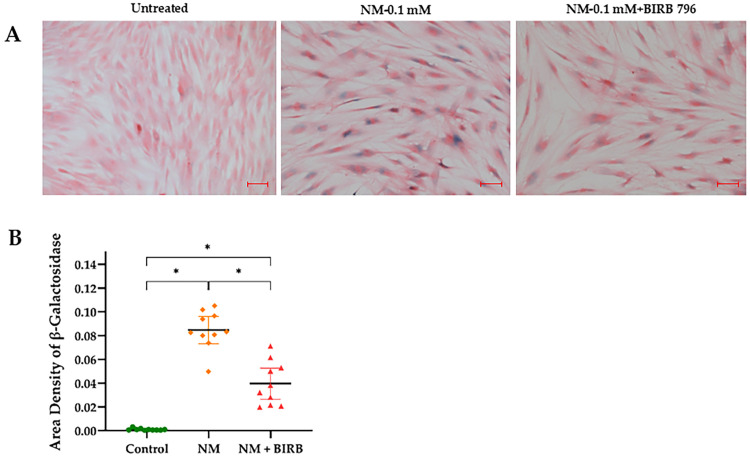
Representative images of β-galactosidase staining in three experimental groups: untreated cells, cells exposed to 0.1 mM nitrogen mustard (NM), and cells exposed to a combination of 0.1 mM NM and BIRB796 after 72 h (scale bar: 50 μm) (**A**). Quantitative analysis of SA-β-gal-positive cells shows that the control group exhibited a significantly lower area density of β-galactosidase compared to both the 0.1 mM NM-treated group and the NM + BIRB group (* *p* < 0.001 for each comparison), with 95% confidence intervals shown. Additionally, the area density of β-galactosidase was significantly higher in the NM-treated group than in the NM + BIRB group (* *p* < 0.001) (**B**).

Β-Galactosidase staining revealed a significant reduction in the number of senescent cells in NM-treated hcMSCs following treatment with the MAPK inhibitor BIRB796, underscoring its potential to prevent NM-induced cellular senescence. The three experimental groups included untreated cells, cells exposed to 0.1 mM NM, and those treated with both 0.1 mM NM and BIRB796, analyzed after 72 h. The control group (M = 0.001; SD = 0.001) exhibited significantly lower β-galactosidase area density compared to the NM-treated group, as confirmed by an independent samples *t*-test, *p* < 0.001. Similarly, the control group showed lower area density compared to the NM + BIRB group, *p* < 0.001. Furthermore, the NM-treated group demonstrated a higher β-galactosidase area density than the NM + BIRB group, *p* < 0.001 (Figure 10).

## 4. Discussion

Nitrogen mustard (NM), a potent alkylating agent, induces significant vision loss and various corneal pathologies, including corneal opacity, neovascularization, epithelial defects, scarring, dry eye, limbal ischemia, and stem cell deficiency. Given the lack of available antidotes, supportive care remains the primary treatment option. A deeper understanding of the mechanisms underlying NM toxicity can pave the way for novel therapies, potentially improving outcomes for severely exposed patients [10,42].

Cellular senescence, marked by irreversible cell cycle arrest and the release of the senescence-associated secretory phenotype (SASP), is a crucial factor in mustard gas toxicity. This mechanism contributes to chronic inflammation and delayed wound healing, prolonging the effects of mustard exposure. Recent studies have demonstrated NM triggers senescence in human dermal fibroblasts and bone marrow-derived mesenchymal stem cells (hBM-MSCs) [21,22,23]. Although this link is well-established, ongoing research aims to further elucidate the signaling pathways involved in NM-induced senescence.

Key senescence markers, such as p16INK4A, p53, and p21CIP1, are significant regulators in this process. Research on veterans exposed to sulfur mustard (SM) has revealed telomere shortening in leukocytes, suggesting a possible connection to cellular senescence [43]. Our findings revealed that both protein and mRNA levels of p16 and p21 were markedly increased at three and fourteen days post-NM exposure, while the proliferation marker Ki67 was significantly reduced.

In the present study, NM induced a dose- and time-dependent transition of human corneal cells (hCCs) from a proliferative state to senescence, evidenced by significantly elevated β-galactosidase staining. These results align with our previous murine model of NM ocular injury, in which NM exposure induced dose-dependent senescence and ocular fibrosis. This underscores the critical role of vesicants in triggering inflammatory cascades that promote senescence and fibrosis [1,21].

In a related study by Rothmiller et al., sulfur mustard was applied to human bone marrow-derived mesenchymal stem cells (MSCs), demonstrating a dose- and time-dependent induction of senescence. The study highlighted significant impairments in MSCs’ proliferative and migratory capacities [23]. Similarly, our results indicate that NM exposure in corneal cells significantly elevates p16INK4A and p21 levels, resulting in diminished proliferative potential. Notably, we observed that the highest NM concentration (0.1 mM) caused the most significant increase in senescence markers, while lower concentrations (0.03 mM and 0.01 mM) produced milder, yet detectable increases in p16INK4A and p21 expression. This dose-dependent effect suggests that NM-induced cellular senescence escalates with increasing exposure levels, likely exacerbating inflammation and tissue remodeling.

The mitogen-activated protein kinase (MAPK) pathway, including p38 and ERK1/2, plays a central role in regulating senescence, cell proliferation, gene expression, and apoptosis [21]. Our study demonstrated that NM exposure activates the MAPK/Akt/AP-1 pathway, leading to the phosphorylation of p38, p44/42, SAPK/JNK, and ERK1/2. Pharmacologic inhibition of p38 MAPK using BIRB796 effectively reduced NM-induced phosphorylation, decreasing the number of senescent cells expressing p16 and p21 while promoting cell proliferation. These findings suggest that MAPK inhibition not only halts senescence progression but also enhances cell survival. Interestingly, the dose–response relationship observed for p16INK4A and p21 expression mirrored the activation of MAPK signaling, with higher NM doses correlating with greater pathway activation and subsequent senescence marker expression.

Excessive activation of MAPK signaling has been implicated in various ocular diseases, including corneal neovascularization, diabetic retinopathy, and advanced forms of age-related macular degeneration [29]. Recent investigations have demonstrated that the MAPK/Akt/AP-1 pathway is activated by the phosphorylation of p38, p44/42, SAPK/JNK, and ERK1/2, leading to inflammation and apoptosis [29]. In our study, both hcMSCs and HCLE cells showed significant increases in the phosphorylation of p44/42 and p38 after 24 h of exposure.

In the present study, as a continuation of our recent works, we hypothesized that MAPK inhibition could be an effective strategy for mitigating the detrimental effects of NM exposure [1,36,42]. Human corneal stromal cells, primary epithelial cells, and a limbal cell line were used to investigate MAPK signaling and the roles of cyclin-dependent kinase 4A (p16INK4A) and the p53/p21WAF1 pathways in the induction of cellular senescence. We demonstrated that inhibiting p38 MAPK with BIRB796 reduced NM-induced phosphorylation in HCLE cells in a dose-dependent manner over 20 h. Furthermore, BIRB796 (0.1 µM) markedly decreased the number of senescent cells expressing p16 and p21 after five days of NM exposure. Notably, lower doses of BIRB796 led to increased Ki67 expression, promoting cell proliferation. These findings suggest that MAPK inhibition not only halts senescence progression but also enhances cell survival, potentially restoring vitality to affected cells.

Multiple known pathways are implicated in NM-induced corneal injuries and may serve as therapeutic molecular targets. Oxidative stress, in particular, plays a significant role in NM-induced corneal injuries, highlighting the potential for antioxidant therapies [17,44]. NM exposure elevates apoptotic markers such as COX-2, MMP-9, and VEGF, contributing to corneal collagen degradation and neovascularization [45,46]. In a mouse model presented by Alemi et al., DNA oxidation was concluded to play a key role in the long-term effects of NM on limbal stem cells. They observed a biphasic ocular injury, primarily affecting the corneal epithelium and anterior stroma [4].

Mesenchymal stem/stromal cells (MSCs) offer a promising therapeutic approach in MK, particularly in reducing corneal opacity and exerting anti-senescence effects [36]. Our recent study showed that mesenchymal stem cell-conditioned media (MSC-CM) significantly improved NM-induced delayed wound healing by reducing reactive oxygen species (ROS) activation and enhancing cell viability [42].

Despite efforts to mitigate or reverse MK, a universally accepted treatment strategy remains elusive. Senolytic agents, which selectively target senescent cells, represent a potential avenue for therapeutic intervention in MK. Small molecules such as rapamycin, dasatinib, and ABT-263 have shown promise in treating ocular disorders linked to senescence. Additionally, therapeutic agents, including dexamethasone, doxycycline, and vitamin D3, have demonstrated efficacy in alleviating NM-induced corneal injuries, further highlighting the need for targeted treatment [2,46].

This study has several limitations, including its narrow focus on NM-induced senescence markers, as only p16 and p21 were evaluated. While we explored the effects of BIRB796, a MAPK p38 inhibitor, further studies are required to assess its influence on additional senescence markers and broader proteins involved in activation pathways. Future investigations should extend to long-term effects beyond the specific time points and concentrations used. Expanding the scope of this research would provide greater insight into the safety and therapeutic potential of MAPK inhibitors. Despite these limitations, our findings provide valuable insights into the therapeutic potential of MAPK inhibitors for treating NM-induced ocular injuries.

## 5. Conclusions

Our findings highlight that nitrogen mustard exposure triggers a dose-dependent induction of cellular senescence in corneal cells. These results align with prior in vivo studies demonstrating enhanced senescence following NM-induced ocular injury. Mechanistically, our data emphasize the involvement of the MAPK pathway in promoting senescence, with MAPK inhibition effectively preventing this process. Inhibiting p38 MAPK not only reduced the expression of senescence markers but also restored the proliferative capacity of corneal cells, demonstrating its potential as a therapeutic strategy. Further research is required to investigate the long-term impact and broader significance of MAPK inhibition.

## Data Availability

All data are available from the corresponding author upon request.
